# Experiences of Young Doctors Working in Rural Nepal

**DOI:** 10.31729/jnma.7504

**Published:** 2022-05-31

**Authors:** Sujata Dahal, Surendra Khanal, Swornima Rijal, Puja Bhandari, Rakshya Pandey

**Affiliations:** 1 Patan Academy of Health Sciences, Lagankhel, Lalitpur, Nepal; 2 Tribhuvan University Teaching Hospital, Maharajgunj, Kathmandu, Nepal; 3 Human Organ Transplant Centre, Dudhpati, Bhaktapur, Nepal; 4 Vayodha Hospital Private Limited, Balkhu, Kathmandu Nepal; 5 Kathmandu Medical College and Teaching Hospital, S, inamangal, Kathmandu, Nepal

**Keywords:** *health*, *health policies*, *rural health*

## Abstract

Medical graduates studying on full and partial scholarships are subjected to a mandatory two years of bonding service program to overcome regional inequalities in the distribution of health workers between rural and urban areas. It might be a challenging journey, but it is crucial for the personal and professional growth of young doctors. Working in places distant from home and medical school can also be difficult, especially when the doctors are not adequately trained. During the bond, young graduates are exposed to a variety of clinical and non-clinical circumstances, which can aid in their maturation.

## INTRODUCTION

The Government of Nepal has implemented the policy of deploying fresh medical graduates to remote areas for two years under a compulsory bonding service program to address regional differences in the distribution of health workers between rural and urban areas. In the context of Nepal, 23% of the doctors were working in the public sector under the mandatory bonded service program.^1^ Between 2012- and 2015, up to five scholarship doctors worked in a district hospital at a time, 36 district hospitals had one or more scholarship doctors, while 34 district hospitals had less than one.^2^ This type of mandatory scholarship bonded program is also prevalent in Australia, India, and Canada.^3^ This article aims to help upcoming graduates understand the practical aspects of the health care system of rural Nepal and guide those who have to fulfill their compulsory binding obligations.

## THE TRANSITION: INTERNSHIP TO INDEPENDENT

While being medical students they completed their medical school at tertiary care centres in the capital city before being appointed to work in the rural areas under full scholarship from the government of Nepal. They have different stories but share a common experience of working in places with scarce resources and the absence of prior training in working in rural areas far from the usual environment.

The internship at the medical college was always supervised by senior residents and consultants so there was a transition from working under guidance to working and making decisions on their own. This was inevitable and required reliance on clinical judgment and maturity which developed gradually.

Weather-wise, the rural places are usually more pleasant and refreshing. However, the differences in the work environment are strikingly noticeable. One of the common difficulties can be the need to travel miles for investigations as trivial as a urine routine examination and culture. The handling of administrative duties can be a new experience. The rural health system relies too much on paramedics and auxiliary staff.

The doctors posted in the rural areas are expected to handle the medico-legal duty which can be a difficult experience with just a basic idea about post-mortem examinations to rely on. The authors were relatively new to injury examinations, sexual assault examinations, and on-site visits with the police as these were not adequately highlighted during their medical schooling. The provision of expert opinions on the cases examined to the local courts can be another unique experience. However, more in-depth and practical knowledge of these subjects during medical school could have been more crucial in the adaptation to the new environment.

## THE PEARLS OF WORKING IN A RURAL AREA

In most rural areas, there is a system in place that provides the doctors and people who work night shifts with an accommodation system. It usually is on a sharing basis, and in many places, there is an assistant for the kitchen. The basic pay for a medical officer is available during the bond and it can be a huge financial relief.

For the patients, the services depend on the centres. In district hospitals where facilities up to cesarean section are available, patients have access to better services than in Primary Health Care Centres (PHCC). Not only that, private laboratories can be present near the hospital offering advanced laboratory investigations. In the PHCC though, management is mostly based on clinical assessment. Most patients in the rural areas present with nonspecific aches and pains requiring nothing but symptomatic relief with paracetamol and proton pump inhibitors.

Small but rewarding experiences in the rural areas can be a more gratifying work experience than working in the tertiary care centres. Leadership and management, coordination among local bodies for the formulation of health policies, and promotion of preventive medicine are some of the aspects that the doctors can grow on as they get a chance to work with full autonomy with the available resources and make the best by following evidence-based protocols.

## CONSTRAINTS OF WORKING IN RURAL AREA

Lack of supervision and guidance, absence of proper electricity, and internet facilities can be the major hindrances for the doctors who have worked only in urban tertiary care centres with no prior experience working in the rural areas. This can affect professional development in the early stage. Also, the practice of using brand names of medicines rampantly rather than using generic names can be problematic.

With the available resources in the rural areas, it is can be difficult to practice according to modern standards. Further, the gap between the knowledge of the most recent protocols and the actual applicability in the rural setting led to the need of practising according to the older guidelines which could also be improved.

## THE PANDEMIC EXPERIENCE

COVID-19 presented as the most out-of-the-blue experience for everyone. In places where the most basic essential medications were hard to find, the plan of adequately managing a pandemic was too ambitious. Social media were reflecting the lack of Personal Protective Equipments (PPE)s, oxygen, and essential medications. However, as primary care doctors, there were disparities in healthcare that were not so evident before the pandemic. And amidst this, the doctors had to work with scarce resources and fear for their own lives, families, and patients.

In the first wave of COVID-19, the authors coordinated with local government bodies and stakeholders to make fever clinics and isolation centres in places with the available resources. Lockdown was ineffective in rural areas because most of the major tasks in the village required gathering. Educating people about COVID-19 and vaccination was important and implementing required strategic measures and the authors did their best to break the chain.

## MESSAGES TO FELLOW JUNIORS

Before processing for the government bonding, the doctors are required to complete their administrative work including a temporary license, database update in the Ministry of Education, and clearance from the university before going to the Department of Health Services (DoHS), because the time required for the process is usually unpredictable. Though not strictly followed, there is a list of available places in the DoHS. Working in a new place, new environment, and with a new team is always a great learning opportunity. However, the following things are worth considering before deciding where the doctors prefer to be working.

- The preference of the community along with the language and the weather
- The hospital itself in regards to the team, accommodation facility, food, transportation, electricity and internet
- Local political dynamics and governing bodies
- Available incentives
- Distance from home
- Plans to prepare for residency exams
- Referral chain

Further, communication with the seniors already working in the centres is crucial to gain an insight into that place. It should be noted that the workplace can be changed with approval from the hospital chief and the DoHS.

With regards to the actual working adaptation, gaining basic ideas about ultrasound examinations and medicolegal issues during internships is essential. Subjects like dermatology, psychiatry, orthopaedics, and Ear Nose Throat (ENT) are not negligible in the periphery. Out-patient and emergency clinical experiences could be more useful than inpatient knowledge but proper clinical acumen before the posting is vital. The doctors might also be requested to home services to local people. As bizarre and inconvenient as it sounds, doing so seemed like a way of survival in a new environment. There is a strong requirement for good communication skills and decision-making capacity while dealing with patients. However, some anxiety and cluelessness at times can be expected but can be overcome with adaptation.

## DISCUSSION

The government-sponsored scholarship is an excellent way to make medical education accessible for candidates from rural backgrounds. Doctors with an upbringing in rural areas are more likely to be retained in rural places.^4^ Exposure to rural communities during medical school and residencies can also help in this. The quality of life in rural areas is inevitably poor compared to urban settings. To lighten the shortage of rural doctors in the United States, strategies employed include offering financial incentives in loan repayment, scholarships, and special curricula for doctors in rural areas during medical school.^5,6^ Decentralising medical education is probably another good way to provide better health services to the people and retain physicians in rural areas. It decentralises specialised manpower so that young doctors get guidance working in rural areas.

The allocation of scholarship doctors during mandatory bonded service programs could have been more scientific if factors such as transparency in the selection process, language barrier, and coordination and communication between concerned authorities were considered. While it is possible that the doctors serve best in the places of their wish but is not very reasonable for most. It is one of the reasons to change the place of work many times during the two years.

In Norway, a graduate receives a random number and is allowed to choose among places based on the numerical order, which sounds more scientific than in Nepal.^7^

The authors realised that it is essential to train for rural practice before being posted there. The primary purpose of the bond is to retain doctors in rural areas as a study shows that training doctors before rural posting increases the chance of staying there for a longer duration.^8^

There are ongoing debates on whether a doctor on bond should be allowed to pursue higher studies and complete them afterward. It is reasonable to let young doctors complete higher education at a young age, but it should also focus on avoiding health centres from becoming devoid of doctors. In the Australian system called Bonded Medical Program, doctors can complete the three-year bond, called the return of service obligations, within 18 years and it does not have to be continuous.^9^

## WAY FORWARD

One can argue that a bonding system that only applies to health professionals is not very equitable while all other fields enjoy the fruits of scholarships free of obligations. While serving the underserved through the bond is not a bad idea, permanent government posts must be fulfilled at any cost. So the authors would like to recommend the DoHS avoid any unnecessary delays in the appointment of graduates to rural posts because stagnancy in skilled manpower is a waste of resources. Serving rural areas can be a very fulfilling experience for young doctors and the process should be as scientific and impartial as possible.
